# “I Feel You!”: The Role of Empathic Competences in Reducing Ethnic Prejudice Among Adolescents

**DOI:** 10.1007/s10964-022-01650-0

**Published:** 2022-07-01

**Authors:** Beatrice Bobba, Elisabetta Crocetti

**Affiliations:** Department of Psychology, Alma Mater Studiorum University of Bologna, Bologna, Italy

**Keywords:** Empathic competences, Ethnic prejudice, Intergroup attitudes, Perspective-taking, Longitudinal

## Abstract

Empathic competences might help adolescents navigate current multicultural societies by supporting harmonious intergroup relations. Yet it is unclear how each component of empathy (empathic concern and perspective-taking) is associated with different dimensions (affective, cognitive, behavioral) of ethnic prejudice. The current study aims to fill this gap. A total of 259 Italian adolescents (*M*_age_ = 15.60, 87.6% female) completed online questionnaires at three time points (i.e., April, May, and October 2021). The results of cross-lagged models indicated that empathic concern was directly and indirectly associated with reduced affective, cognitive, and behavioral ethnic prejudice, while perspective-taking was linked to increases in cognitive and one facet of behavioral (i.e., lower contact willingness) prejudice. Furthermore, the prevalence of affect over cognition was found, with the affective component of both empathic competences (i.e., empathic concern) and ethnic prejudice exerting the strongest influence on the cognitive ones.

## Introduction

Migration flows and geopolitical changes have brought adolescents to live in increasingly multicultural societies and interact with peers and adults of different ethnic backgrounds (Bagci & Rutland, 2019). However, prejudice of ethnic majority adolescents against ethnic minorities and immigrants is still a matter of concern (Crocetti et al., 2021). In this regard, young people are generally more tolerant toward social groups that have traditionally been marginalized (such as sexual minorities), but they are less accepting of the immigrant groups compared to older generations (Janmaat & Keating, 2019). Therefore, a core issue is to understand which factors could reduce adolescents’ prejudice in order to promote more inclusive relations in multicultural societies. While empathy can play a key role in lessening negative intergroup attitudes (Aboud & Amato, 2002; Rutland & Killen, 2015), the ways in which affective and cognitive components of empathic competences are developmentally related to distinct facets of prejudice are still largely unknown. Therefore, this study took a multidimensional approach to examine longitudinal associations between different dimensions of empathic competences and multiple components (i.e., affective, cognitive, and behavioral) of ethnic prejudice in adolescence. To unravel these links is of utmost importance to develop evidence-based interventions aimed at improving the quality of relations among adolescents of different ethnic groups.

### Empathic Competences: The Interplay of Empathic Concern and Perspective-Taking

In adolescence, youth undergo significant changes in personality and relationships and develop more refined cognitive, social, moral, and emotional competences (Meeus, 2019). These crucial advancements influence adolescents’ views of themselves and others, which progressively stabilize into mature attitudes and approaches to society and diversity (Crocetti et al., 2021). A key individual skill that has been linked to positive intergroup experiences and adjustment is empathic competences.

As a trait, empathic competences refer to the individuals’ general disposition of engaging in an affective and cognitive response after the apprehension of someone else’s emotional state (Malti & Ongley, 2014). Experiencing emotions consistent with those of a target person (the so-called parallel empathy) often results in *empathic concern*, which involves other-oriented feelings of sorrow and sadness for the person’s unfavorable condition and represents the affective component of empathic competences (Eisenberg et al., 2006). The cognitive component is *perspective-taking*, which implies the ability to understand and take on the point of view of a target person (Van der Graaff et al., 2020).

Although interdependent, empathic concern and perspective-taking are distinct constructs. Besides relying on different neural bases (Stietz et al., 2019), they follow specific developmental trajectories (Van der Graaff et al., 2020). While most studies highlighted an increase in perspective-taking abilities (e.g., Miklikowska et al., 2011), research on the development of empathic concern reported mixed findings (for a review, see Van der Graaff et al., 2020). Furthermore, empathic concern and perspective taking have shown unique associations with several outcomes (e.g., prosocial behavior, conflict resolution; Van der Graaff et al., 2018; Van Lissa et al., 2016) in adolescence.

From a theoretical standpoint, emotional contagion (or parallel empathy) is expected to precede the cognitive appraisal of the other person’s point of view (i.e., perspective-taking), which in turn causes empathic concern (Batson, 2009; Decety, 2005). Empirical research testing these theoretical assumptions is still scarce, and findings are mixed. For instance, reciprocal longitudinal associations were found between empathic concern and perspective-taking among Swedish (Miklikowska, 2018) and Dutch adolescents (Van der Graaff et al., 2018), with the former predicting subsequent levels of the latter and vice versa. However, when separating within- and between-person variance, the effect of empathic concern on perspective-taking was stronger than its reverse (Miklikowska, 2018). In line with this, another study that followed adolescents longitudinally only found a significant effect of empathic concern on subsequent levels of perspective-taking (van Lissa et al., 2014), highlighting the key role of the affective component.

Taken together, there is preliminary support for the prevalence of affect over cognition in the interplay of the two dimensions of empathic competences. The current study aimed to contribute to the literature by unraveling the longitudinal associations between empathic concern and perspective-taking. Additionally, it examined the unique associations of each dimension of empathic competences with different components of ethnic prejudice.

### Ethnic Prejudice and its Components

Ethnic prejudice can be defined as “any attitude, emotion, or behavior” (Brown, 2011, p. 7) that people hold against one or more ethnic outgroup(s). Therefore, prejudice implies multiple components, which are facets of the same general orientation. *Affective prejudice* refers to negative feelings and evaluations (i.e., disliking) elicited by one or more ethnic group(s). In contrast, *cognitive prejudice* implies a set of usually negative beliefs and opinions (i.e., stereotypes) about members of the outgroup. Additionally, prejudice also includes *behavioral tendencies* (e.g., avoidance, discrimination) that usually express underlying cognitions and affects (Brown, 2011; Cuddy et al., 2007). This three-dimension model aligns with the traditional Affect-Cognition-Behavior (ABC) models of attitudes (Eagly & Chaiken, 1993; Rosenberg & Hovland, 1960).

It has long been debated whether a predominant component of prejudice over the others could be identified, with the former leading the organization of the latter (Hamilton & Mackie, 1993). One approach considers cognitive processes (i.e., categorization, salience, biases) as pivotal driving forces influencing emotional reactions and behavioral tendencies toward a target outgroup. For instance, the classic experiments on the minimal group paradigm (Tajfel et al., 1971) showed that mere categorization in different groups drive preference for one’s own group and discrimination against members of other groups (Bigler & Liben, 2007). Additionally, the stereotype content model (Fiske et al., 2002) highlighted that cognitive prejudice (i.e., stereotypes) stemming from social categorization and appraisal processes lead to emotional reactions (i.e., the affective component of prejudice) toward the target groups. Additionally, these theoretical approaches have considered affective prejudice a key antecedent of behavioral tendencies (Dovidio et al., 2004) and a mediator in the association between cognitive and behavioral prejudice (Cuddy et al., 2007). Conversely, other approaches underline the precedence of affect over cognition and behavior (e.g., Zajonc, 1998). For instance, intergroup emotions (i.e., emotions stemming from self- and other-categorization into relevant social groups; Smith & Mackie, 2008), rather than stereotype knowledge, have been found to mediate the association between intergroup contact and affective and cognitive components of prejudice (Miller et al., 2004). Overall, different theoretical approaches and findings within each line of research have provided support for the leading role of one or the other dimension of prejudice.

However, to the extent of our knowledge, no previous study has empirically tested the longitudinal associations between the affective and cognitive components of ethnic prejudice and how these dimensions might in turn influence their behavioral counterpart in adolescence. Affective, cognitive, and behavioral prejudice have been found to display different developmental trajectories (Bobba et al., 2022) and levels of rank-order stability in adolescence (for a meta-analysis, see Crocetti et al., 2021). Therefore, accounting for all three components is crucial to shed light on how ethnic prejudice develops and organizes during this life stage, considering youth’s concurrent advancements in cognition, morality, and emotion regulation. Are affective, cognitive, and behavioral prejudice reciprocally associated? Or is it possible to identify a dimension driving changes in the other(s)? More importantly, do empathic competences impact these dimensions of prejudice differently?

### Empathic Competences and Ethnic Prejudice: What is Known and Open Questions

Being able to take on the perspective of other people might increase perceived similarities between self and others and reduce the dichotomous view of “Us vs. Them”, which is at the core of negative intergroup attitudes and experiences (Tajfel & Turner, 1979; van Bommel et al., 2020). Moreover, empathic concern could increase interest in others’ well-being and motivate direct altruistic behaviors to alleviate someone else’s unfavorable conditions (De Waal, 2008). Experimental research has generally confirmed these theoretical assumptions. For instance, inducing participants to take on the perspective of an outgroup member increased their liking of the target outgroup (e.g., Shih et al., 2009) and improved intergroup attitudes (e.g., Dovidio et al., 2004; Vescio et al., 2003) and behaviors (e.g., Adida et al., 2018; Sierksma et al., 2014). Additionally, a recent meta-analysis on prejudice reduction interventions has highlighted the effectiveness of empathy training, which appears to be the second most effective intervention to reduce prejudice after direct intergroup contact (for a review, see Beelmann & Heinemann, 2014).

The Empathy-Attitudes-Action model (Batson et al., 1997, 2002) provides a useful framework to understand the associations between empathic competences, prejudicial attitudes, and prosocial tendencies in intergroup contexts. Specifically, this four-step model implies that (a) people assume the perspective of others and develop other-oriented feelings, (b) which increase the valuing of others’ well-being, and in turn (c) translates into more positive attitudes, that (d) underlie and support more prosocial intentions (Batson et al., 2002). Experimental findings with adults (e.g., Batson et al., 2002) and children (Taylor & Glen, 2020) have generally confirmed this model: eliciting empathic responses causes a reduction in prejudice which in turn increases helping intentions toward the outgroup.

These associations have also been tested in a recent longitudinal study with adolescents (Taylor & McKeown, 2021). Trait empathy was associated with more positive feelings toward the outgroup, higher helping intentions, realistic helping, and collective action over time, with attitudes mediating the link between empathic competences and prosocial actions. However, this longitudinal study did not distinguish the effect of affective and cognitive empathic competences, leaving an open question about the unique role played by these components. On this line, bidirectional longitudinal associations between empathic concern, perspective-taking, and (cognitive) prejudice emerged among adolescents (Miklikowska, 2018). However, when examining within-person changes (while controlling for between-person differences), perspective-taking was directly associated with changes in prejudice, while empathic concern indirectly influenced subsequent prejudice levels via its effect on perspective-taking (Miklikowska, 2018).

These longitudinal findings confirm the role of trait empathic competences in reducing ethnic prejudice. However, they have mostly examined the associations of empathic competences with one form of ethnic prejudice at a time and have not accounted for its multifaceted nature. Therefore, prior studies do not provide a comprehensive understanding of how empathic concern and perspective-taking might differentially influence the affective, cognitive, and behavioral components of ethnic prejudice. Such influence might be specifically powerful between dimensions that pertain to shared psychological spheres, mainly the affective and cognitive ones. That is, empathic concern (i.e., the affective component of empathic competences) might exert the strongest influence on affective ethnic prejudice, while perspective-taking (i.e., the cognitive component of empathic competences) might be more strongly associated with cognitive ethnic prejudice over time. Additionally, empathic concern might also influence the behavioral component of prejudice, as intergroup behaviors are usually guided by affective mechanisms (Dovidio et al., 2010). This study aimed to fill the gaps outlined above by testing this dimension-matching effect and providing a more nuanced understanding of the longitudinal reciprocal associations across and between multiple components of empathic competences and ethnic prejudice.

## Current Study

Extensive research has highlighted that empathy can lessen prejudice over time but has neglected to account for the multifaceted nature of both empathic competences (i.e., empathic concern and perspective-taking) and prejudice (i.e., affective, cognitive, and behavioral). Thus, the purpose of the current study was threefold. First, it aimed to test the predominant role of the affective dimension of empathic competences and ethnic prejudice in leading changes in the other component(s) of each construct. Second, this study examined the reciprocal direct associations of empathic competences and ethnic prejudice over time to test a dimension-matching hypothesis. That is, the affective (i.e., empathic concern and affective prejudice) and cognitive (i.e., perspective taking and cognitive prejudice) components of both constructs were expected to be strongly associated with each other. Third, mainly adopting an exploratory approach, this study aimed to test possible indirect associations across and between empathic competences and prejudice components. For instance, perspective-taking could mediate the association between empathic concern and cognitive ethnic prejudice, whereas empathic concern might be indirectly associated with cognitive prejudice via its effects on affective prejudice.

## Methods

### Participants

Participants in this three-waves longitudinal study were 259 adolescents (*M*_age_ = 15.60, *SD*_age_ = 0.63, 87.6% females) attending the 1st and 2nd year of a high school located in the North-East of Italy. Since the focus was on prejudice against ethnic minorities, only Italian adolescents were included in the current study (i.e., youth of immigrant descent were excluded). At baseline, most students reported their parents were married (73.4%), while 22.4% reported their parents were separated or divorced. Regarding parents’ educational level, most of the adolescents’ mothers (51.8%) had a medium educational level (i.e., high school diploma), while some (29.3%) had a high (i.e., university degree or higher) and a few (18.9%) a low (i.e., up to middle school diploma) educational level. As for fathers, most of them (47.9%) had a medium educational level, followed by those with low (27.4%) and high (24.7%) educational level.

Most adolescents participated in all three assessments (71.8%), while almost all of them (95.6%) in at least two assessments. Within each assessment, completion rate of the questionnaires was very high (99.6% at T1 and T3, 100% at T2). Therefore, missingness was mostly due to participants not filling out the questionnaire, mainly because they were not in school on the day of data collection. The Little’s (1988) Missing Completely at Random (MCAR) test conducted on the study variables yielded a normed χ^2^ (χ^2^/df = 340.339/332) of 1.025, indicating that data were likely missing completely at random. Therefore, the total sample of 259 participants was included in the analyses, and missing data were handled with the Full Information Maximum Likelihood (FIML) procedure available in M*plus* (Kelloway, 2015).

### Procedure

The present study was approved by the Ethics Committee of the Alma Mater Studiorum University of Bologna (Italy). Permission from the school principal and active consent from parents and adolescents were obtained prior to data collection. Participation in the study was voluntary, and students were informed they could withdraw their consent at any time. Data collection consisted of three waves spanning across two academic years. The first two waves were one month apart in April (T1) and May (T2) 2021, while the last follow-up was at the beginning of the following academic year (T3: October 2021). Due to the COVID-19 pandemic, data were collected during remote (T1), hybrid (T2), and in-presence (T3) school hours. At all waves, participants completed online questionnaires on Qualtrics and were required to create a personal code to ensure confidentiality and link their responses over time. An extract of the study materials can be retrieved at: https://osf.io/k8ypz/.

### Measures

#### Demographics

Participants’ socio-demographic information (i.e., age, gender, nationality, family composition, living conditions, parents’ educational level) were collected at T1.

#### Empathic concern

The affective component of empathic competences was assessed using the empathic concern subscale of the Interpersonal Reactivity Index (IRI; Davis, 1980; Italian validation by Albiero et al., 2006). This subscale comprises seven items (e.g., “I often have tender, concerned feelings for people less fortunate than me”) and participants were asked to rate their agreement on a 5-point Likert scale (from 1 “*completely disagree*” to 5 “*completely agree*”). Cronbach’s Alphas were 0.68, 0.73, 0.76 at T1, T2, and T3, respectively.

#### Perspective-taking

The perspective-taking subscale of the Interpersonal Reactivity Index (IRI; Davis, 1980; Italian validation by Albiero et al., 2006) was used to evaluate the cognitive component of empathic competences (seven items; e.g., “I sometimes try to understand my friends better by imagining how things look from their perspective”). Participants rated their agreement with each statement using a 5-point Likert scale (from 1 “*completely disagree*” to 5 “*completely agree*”). Cronbach’s Alphas were.74, 0.79, 0.80, at T1, T2, and T3, respectively.

#### Affective prejudice

The affective component of prejudice was assessed using the Feeling thermometer (Haddock et al., 1993; for the Italian version, see Albarello & Rubini, 2011), asking participants to rate how much they like different outgroups (i.e., Romanians, Albanians, Moroccans, Chinese, and Ukrainians were chosen since they are the most represented groups of foreigners in Italy according to ISTAT, 2020) on a scale from 0° (*not at all*) to 100° (*very much*). The scale was reversed to simplify the interpretation of results, with higher scores indicating higher prejudice. A total affective prejudice score was computed using the mean level of liking expressed for these different outgroups. Cronbach’s Alphas were 0.97, 0.98, 0.96 at T1, T2, and T3, respectively.

#### Cognitive prejudice

To evaluate the cognitive component of prejudice, nine items (e.g., “Foreign people should be marginalized in Italian society”) were adapted from Brown et al. (2008). Participants rated their agreement on a 5-point Likert scale (from 1 “*completely disagree*” to 5 “*completely agree*”). Cronbach’s Alphas were 0.88, 0.93, 0.90 at T1, T2, and T3, respectively.

#### Behavioral prejudice

The behavioral dimension of prejudice was assessed using two different scales. The contact willingness scale (Titzmann et al., 2015) consists of three items asking participants whether they would enjoy different interactions with outgroup members (e.g., “I can imagine having immigrant friends”). Additionally, six items were selected from the outgroup helping intentions scale (Johnston & Glasford, 2018), asking respondents whether they would help a target outgroup person in need (e.g., “You give directions to a foreign stranger who appears to be lost”). Participants rated their answers using a 5-point Likert scale (from 1 “*completely disagree*” to 5 “*completely agree*”). The responses were recoded, so that higher scores were indicative of higher behavioral prejudice (i.e., less contact willingness and intentions to help). Cronbach’s Alphas were 0.72, 0.77, 0.75 for contact willingness and 0.70, 0.77, 0.76 for helping intentions, at T1, T2, and T3, respectively.

## Results

### Preliminary Analyses

Descriptive analyses were computed using IBM SPSS Version 23.0 for Windows. Means, standard deviations, and correlations among study variables are reported in Table S1 of the Supplemental Materials. All the remaining analyses were conducted in M*plus 8.6* (Muthén & Muthén, 1998–2017), using Maximum Likelihood Robust (MLR) estimator (Satorra & Bentler, 2001). Analyses codes and outputs can be retrieved from https://osf.io/k8ypz/. As a preliminary step, longitudinal measurement invariance was examined separately for all study variables. Metric invariance could be established for all constructs (see Table S2 of the Supplemental Materials), and therefore we could proceed with testing the main hypotheses.

### Cross-Lagged Associations of Empathic Competences and Ethnic Prejudice

**Fig. 1:**
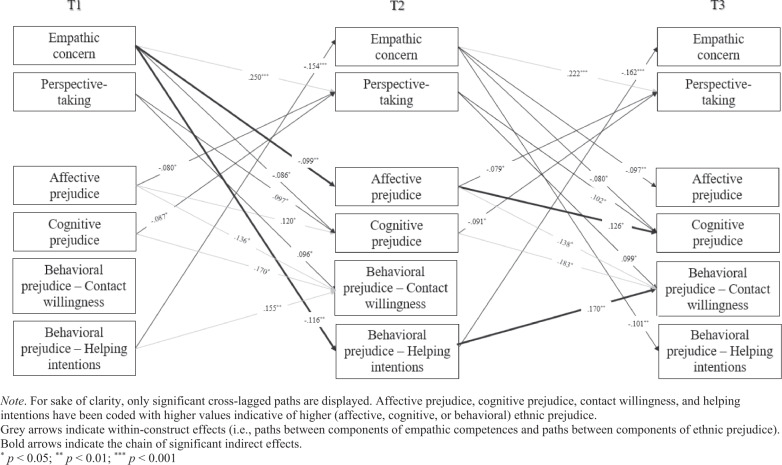
Significant standardized results of the cross-lagged model.

The current study aimed to disentangle reciprocal longitudinal associations between the affective (i.e., empathic concern) and cognitive (i.e., perspective-taking) dimensions of empathic competences and the affective, cognitive, and behavioral dimensions of ethnic prejudice. To this end, a cross-lagged panel model with observed variables was tested. First, an unconstrained model (M1) with cross-lagged paths between empathic competences and dimensions of ethnic prejudice was estimated, controlling for: (a) stability or autoregressive paths (i.e., T1 → T2, T2 → T3, T1 → T3), and (b) within-time correlations among all variables (i.e., correlations among variables at T1, and correlated changes at T2 and T3). This model showed a very good fit (Table 1), based on a combination of the following indices (Byrne, 2012): the Comparative Fit Index (CFI) and the Tucker-Lewis Index (TLI), with values higher than 0.90 and 0.95 indicative of acceptable and very good fit, respectively; and the Root Mean Square Error of Approximation (RMSEA) and the Standardized Root Mean Residual (SRMR) with values below 0.08 and 0.05 indicative of an acceptable and very good fit, respectively. Additionally, the RMSEA’s 90% confidence interval’s upper bound lower than 0.10 indicates an acceptable model fit (Chen et al., 2008). Next, to identify the most parsimonious model of reciprocal associations, a model (M2) with cross-lagged paths fixed to be equal across waves (i.e., T1 → T2 paths constrained to be equal to T2 → T3 paths) was tested and compared against the unconstrained one, and a model (M3) with fixed cross-lagged paths and fixed correlated changes (i.e., within-time correlations at T2 and T3) was tested and compared against M2. Differences between models were identified if at least two of the following criteria were met: a Δχ_SB_^2^ significant at *p* < 0.05 (Satorra & Bentler, 2001), ΔCFI ≥ −0.010, and ΔRMSEA ≥ 0.015 (Chen, 2007). Results indicated that time-invariance of both cross-lagged paths and correlated changes could be established (Table 1). Thus, the most parsimonious model (M3) was retained as the final one. Stability paths are reported in Table 2 together with within-time correlations (T1) and correlated changes (T2 and T3). Significant cross-lagged paths are reported in Fig. 1.

**Table 1:** Cross-lagged panel model: Model fit indices and model comparison

Models	Model fit						Model comparisons			
	χ_SB_^2^	df	CFI	TLI	SRMR	RMSEA [90% CI]	Models	Δχ_SB_^2^	ΔCFI	ΔRMSEA
Unconstrained (M1)	44.210	30	0.992	0.963	0.016	0.043 [0.006, 0.068]				
Cross-lagged paths fixed (M2)	84.814	60	0.986	0.968	0.032	0.040 [0.017, 0.059]	M2-M1	40.803 (30)	−0.006	−0.003
Cross-lagged paths and within time correlations fixed (M3)	115.682	75	0.977	0.958	0.041	0.046 [0.028, 0.062]	M3-M2	29.100 (15)*	−0.009	0.006

Note. *χ*_*SB*_^*2*^ Satorra-Bentler scaled chi-square, *df* degree of freedom, *CFI* comparative fit index, *TLI* Tucker-Lewis index, *SRMR* standardized root mean square residual, *RMSEA* root mean square error of approximation, *CI* confidence interval, Δ change in the parameter

*p < 0.05; **p < 0.01; ***p < 0.001

**Table 2:** Standardized results of the cross-lagged panel model

Stability paths	T1 → T2	T2 → T3	T1 → T3
Empathic concern	0.614***	0.457***	0.217**
Perspective-taking	0.504***	0.449***	0.193***
Affective prejudice	0.709***	0.594***	0.189*
Cognitive prejudice	0.504***	0.288**	0.353***
Behavioral prejudice - Contact willingness	0.396***	0.214*	0.136*
Behavioral prejudice - Helping intentions	0.623***	0.395***	0.235**

Correlations	T1	T2	T3
Empathic concern ↔ Perspective-taking	0.299***	0.289***	0.315***
Empathic concern ↔ Affective prejudice	−0.241***	−0.113**	−0.129**
Empathic concern ↔ Cognitive prejudice	−0.110	−0.139*	−0.155**
Empathic concern ↔ Contact willingness	−0.191**	−0.201***	−0.185***
Empathic concern ↔ Helping intentions	−0.377***	−0.321***	−0.281***
Perspective-taking ↔ Affective prejudice	−0.202***	−0.124**	−0.154**
Perspective-taking ↔ Cognitive prejudice	−0.251***	−0.115*	−0.139*
Perspective-taking ↔ Contact willingness	-0.231***	−0.166**	−0.167**
Perspective-taking ↔ Helping intentions	−0.321***	−0.177***	−0.169***
Affective prejudice ↔ Cognitive prejudice	0.530***	0.266***	0.338***
Affective prejudice ↔ Contact willingness	0.437***	0.223***	0.235***
Affective prejudice ↔ Helping intentions	0.397***	0.175**	0.175**
Cognitive prejudice ↔ Contact willingness	0.580***	0.488***	0.501***
Cognitive prejudice ↔ Helping intentions	0.513***	0.245***	0.239***
Contact willingness ↔ Helping intentions	0.501***	0.392***	0.317***

*p < 0.05; **p < 0.01; ***p < 0.001

To address the first aim of the study, within-construct associations were examined. The hypothesis of affect prevalence was confirmed by the data. Results showed that empathic concern was positively associated with perspective-taking over time, while the opposite was not true, and affective prejudice predicted subsequent levels of its cognitive counterpart but not the other way around. As for the two behavioral components, contact willingness was influenced by all the other dimensions of ethnic prejudice over time, while helping intentions was not.

To tackle the second aim of the study, associations between empathic competences and ethnic prejudice over time were examined. The dimension-matching hypothesis was partially confirmed by the data. Specifically, empathic concern was negatively associated with affective and one measure of behavioral (i.e., low helping intentions) ethnic prejudice over time. However, empathic concern was also, albeit marginally (*p* = 0.046), significantly associated with lower cognitive prejudice over time. Thus, while the association between empathic and affective prejudice confirmed the dimension-match hypothesis, additional paths highlighted a more nuanced pattern of associations. Furthermore, perspective-taking was positively associated with cognitive and one measure of behavioral (i.e., low contact willingness) ethnic prejudice. Regarding associations in the opposite direction, ethnic prejudice dimensions were associated with lower empathic competences over time. Specifically, affective and cognitive prejudice contributed to lower perspective-taking, while behavioral prejudice (i.e., low helping intentions) was negatively associated with empathic concern.

Finally, in line with the third aim of the study, taking an exploratory approach, all possible indirect effects were examined using the indirect command procedure available in M*plus*. Two significant indirect effects were found (see results displayed in Fig. 1, bold arrows). First, empathic concern at T1 was associated with lower levels of cognitive prejudice at T3 via affective prejudice at T2 (standardized indirect effect = −0.012 [−0.024, 0.000], *p* = 0.043). Additionally, empathic concern at T1 was associated with lower levels of contact (un)willingness at T3 via helping intentions at T2 (standardized indirect effect = −0.025 [−0.037, −0.002], *p* = 0.026). Contrary to our expectations, perspective-taking at T2 did not mediate the relation between empathic concern at T1 and ethnic prejudice dimensions at T3.

### Sensitivity Analyses

As ancillary sensitivity analyses, the same cross-lagged panel model was tested including participants’ gender and their parents’ educational level as covariates. These demographic variables have been previously associated with adolescents’ prejudice levels (Rekker et al., 2015; Weber, 2019) and differences in levels and developmental trends of empathic competences (Van der Graaff et al., 2014). Except for a couple of paths (which were marginally significant in the original model and lost significance when accounting for covariates), the model was largely replicated, highlighting the robustness of the findings. Results of these sensitivity analyses are reported in Tables S3a and S3b of the Supplementary Materials.

## Discussion

Adolescence is a crucial phase for the development of more sophisticated cognitive, moral, and social competences (Meeus, 2016). Such advancements are the foundations upon which youth form specific views of themselves, others, and society (Crocetti et al., 2016; 2021). Therefore, it is crucial to understand which individual competences, such as the empathic ones, might effectively support positive intergroup experiences and enhance adolescents’ adjustment in multicultural societies (Titzmann & Jugert, 2019). The current study advanced extant knowledge by taking a multidimensional perspective to disentangle the unique associations between each component of empathic competences (i.e., empathic concern and perspective-taking) and multiple dimensions (i.e., affective, cognitive, and behavioral) of ethnic prejudice among adolescents. Overall, these findings highlighted the predominant role of affective over cognitive processes, and the protective role of empathic concern in preventing the development of negative intergroup emotions, attitudes, and behaviors. Such knowledge is crucial not only to extend the theoretical understanding of these associations, but also to design evidence-based interventions aimed at equipping adolescents with useful competences to approach diversity and support harmonious intergroup relations.

### What Drives Change? The Predominant Role of Affective Components

The first goal of the present research was to test the predominant role of affect over cognition in influencing the other dimension(s) of empathic competences and ethnic prejudice. Results confirmed this hypothesis. Regarding empathic competences, empathic concern was found to influence subsequent levels of perspective-taking, while the reverse was not true. This finding aligns with previous research on adolescents (van Lissa et al., 2014), highlighting the leading role of empathic concern over perspective-taking and confirming the precedence of affect over cognition in understanding others’ experiences. This is also in line with research on the developmental trajectories of empathic competences, which consistently highlighted higher levels of empathic concern compared to perspective-taking among adolescents (Van der Graaff et al., 2014). As the neural bases of empathic concern have been found to develop early on (Singer, 2006), the affective dimension of empathic competences displays higher stability than its cognitive counterpart (van Lissa et al., 2014), which might explain the unidirectional association found in the current study. Following a “top-down” approach (De Waal, 2007), cognitive processes such as perspective-taking stem from and are driven by affective ones. Therefore, feelings of sorrow and concern for other people’s misfortunes might induce adolescents to understand the experiences of others better by taking their perspective.

Similarly, affective prejudice was associated with higher levels of cognitive prejudice over time, but not the other way around. That is, the affective reactions towards members of the outgroup inform and orient subsequent stereotypes and beliefs against them, which then inform behavioral tendencies in intergroup contexts. The present study considered two different forms of behavioral ethnic prejudice: low willingness of contact with members of the foreign group and low intentions to help foreign people in need, which displayed very different patterns of associations. The former was influenced by both affective and cognitive prejudice, while the latter appeared to be influenced by empathic concern only. It could be argued that low contact willingness represents the behavioral conversion of adolescents’ ethnic prejudice, while helping intentions might tap into the general domain of prosociality rather than being a measure of behavioral prejudice per se. Prior research has confirmed the strong associations between empathic competences and prosocial behaviors (e.g., Metzger et al., 2018; Van der Graaff et al., 2018; for a review, see Malti et al., 2021), and might therefore explain the unique inverse reciprocal links observed in the current study among empathic concern and low helping intentions.

### Empathic Competences and Prejudice: The Protective Role of Empathic Concern

Regarding the second aim of the present study, results showed reciprocal longitudinal associations between empathic competences and ethnic prejudice, although the dimension-matching effect was only partially supported. Specifically, empathic concern was indeed directly associated with lower levels of affective prejudice and of one form of behavioral prejudice (i.e., low helping intentions), but it was also linked, to a lesser extent, to lower levels of cognitive prejudice. These different effects partially support the dimension-matching hypothesis, that is empathic concern (i.e., the affective dimension of empathic competences) tackles the affective component of prejudice more directly because they both tap into affects and emotions. These findings highlight the crucial role of empathic concern in reducing affective, cognitive, and behavioral components of ethnic prejudice.

Moving into perspective-taking, our findings displayed a complex pattern of concurrent and longitudinal associations with ethnic prejudice. Concurrent associations were in line with a wide literature (e.g., Adida et al., 2018; Miklikowska, 2018), showing that higher levels of perspective-taking were linked with lower affective, cognitive, and behavioral ethnic prejudice. In contrast, cross-lagged paths indicated that perspective-taking was associated with higher levels of cognitive prejudice and of the other form of behavioral prejudice (i.e., low contact willingness) over time. These latter longitudinal findings could be interpreted in light of the literature on cognitive empathy and bullying. In fact, the ability to take on the perspective of others and understand their point of view might serve either altruistic or egoistic purposes (Eisenberg et al., 1991). This means that perspective-taking skills do not automatically imply an increased interest in other people’s well-being and subsequent prejudice reduction, as argued by the Empathy-Attitudes-Action model (Batson et al., 1997, 2002). Adolescents might understand the perspective of others but still maintain their negative feelings and cognitions about them and, even worse, engage in bullying and victimization (Bayram Özdemir et al., 2020). On the contrary, those who display higher empathic concern might be more sensitive to other people’s sufferings, display stronger awareness of the negative consequences of discrimination against minorities, and therefore show lower ethnic prejudice.

### Confirming the Precedence of Affect over Cognition: Longitudinal Mediations

Regarding the third and final aim of the present study, the longitudinal indirect associations across and between empathic competences and ethnic prejudice dimensions confirmed the precedence of affect over cognition. First, empathic concern was associated with reduced cognitive prejudice via its effect on affective prejudice, while the reverse direction (i.e., from empathic concern to affective prejudice via cognitive prejudice) was not supported by the results. Additionally, empathic concern was indirectly associated with decreases in low contact willingness, via its effects on helping intentions. When the same associations were tested with perspective-taking, no significant indirect effect was found.

Moreover, perspective-taking did not mediate the associations between empathic concern and different dimensions of ethnic prejudice. This finding is in contrast with previous longitudinal research (Miklikowska, 2018). However, it should be noted that in the current study perspective-taking alone was associated with increased prejudice, whereas empathic concern was found to reduce prejudice over time. Thus, these opposite effects might have counteracted each other, resulting in no significant indirect effect from empathic concern to prejudice via perspective-taking.

Overall, these findings speak for the precedence of affective processes over and above the cognitive ones. Immediate affective and emotional reactions arising in intergroup contexts (i.e., intergroup emotions; Smith & Mackie, 2008) might first inform affective responses (i.e., disliking members of minority ethnic groups), which in turn drive their cognitive counterpart (i.e., negative beliefs and stereotypes against minorities). Disentangling associations between and across empathic competences and ethnic prejudice allows not only for advancements in the theoretical understanding of these phenomena, but also for planning evidence-based interventions aimed at supporting adolescents’ intergroup relations.

### Practical Implications

Overall, this study has important practical implications. First of all, it highlighted the prevalence of affective over cognitive processes and the crucial role played by empathic concern in reducing ethnic prejudice. Previous intervention and experimental studies (e.g., Adida et al., 2018; Shih et al., 2009) have generally focused on the perspective-taking component to support more positive intergroup attitudes and relationships. Although it might be easier to induce participants to take on the point of view of others rather than to sympathize with them (van Lissa et al., 2014), empathic concern seems to be the key to breaking the vicious dichotomous view of “Us vs. Them”. Therefore, training adolescents in socio-emotional skills (such as empathic concern) might prove effective for supporting positive intergroup relations in current multicultural societies. To maximize their effectiveness, intervention programs should be developmentally sensitive to students’ starting level of empathic competences, their strengths and difficulties (Malti et al., 2016).

Moreover, as affective prejudice appears to influence cognitions and behaviors in intergroup contexts in adolescence, interventions should also tackle this dimension first, which might have a snowball effect on the others. For instance, previous studies have found that positive intergroup contact experiences work very well in reducing affective prejudice (e.g., Aberson, 2015; Tropp & Pettigrew, 2005). Therefore, interventions based on contact might be a useful tool to reduce ethnic prejudice by tackling its affective dimension first and, consequently, its cognitive and behavioral manifestations.

### Limitations and Suggestions for Future Research

This study contributed to disentangling longitudinal associations across and between multiple dimensions of empathic competences and ethnic prejudice in adolescence and has important theoretical and practical implications. However, some limitations should be considered. First, participants were not equally distributed based on gender, with females comprising more than two-thirds of the sample. Previous research confirms that male and female adolescents report different levels and developmental trends of empathic competences (e.g., Van der Graaff et al., 2014; for a review, see Meeus, 2019) and ethnic prejudice (Rekker et al., 2015), as also emerged from the sensitivity analyses (i.e., when gender was included it was related to increases in empathic concern over time). Therefore, current findings might have been influenced by the sample’s gender imbalance, and generalization should be made with caution.

Additionally, using a traditional cross-lagged panel model did not allow for the distinction of between- and within-person variance (Hamaker et al., 2015). However, it was not possible to test the same reciprocal associations between empathic competences and ethnic prejudice using the random intercept cross-lagged panel model because of convergence issues, which have been highlighted in previous research (Orth et al., 2021; Usami et al., 2019).

Finally, the current three-waves study covered a relatively short period (i.e., six months). This limited the possibility to highlight significant developmental changes in empathic competences and ethnic prejudice, which might occur at a slower pace. Thus, future studies could benefit from including multiple yearly assessments to examine how these longitudinal associations unfold over the course of a longer time span.

## Conclusion

Research has supported the notion that feeling concern for and assuming the perspective of others might be useful competences to overcome the dichotomous view of “Us vs. Them” that is at the core of heinous forms of ethnic prejudice. However, prior studies have neglected to consider the multifaceted nature of both empathic competences and ethnic prejudice. This study took a step forward uncovering the longitudinal associations across and between different dimensions of these constructs. Looking at within-construct associations, the prevalence of affect over cognition was found by showing that the affective component of both empathic competences (i.e., empathic concern) and ethnic prejudice exerted the strongest influence on the cognitive ones. Additionally, examining between-construct associations, this study found empathic concern to reduce all forms of prejudice either directly or indirectly, while perspective-taking was linked to increases in prejudice over time. These findings might inform future interventions to foster adolescents’ interpersonal and intergroup competences and support harmonious relations in current multicultural societies.

## Supplementary Information

Supplementary Materials
