# Supplementary material for: “I Feel You!”: The Role of Empathic Competences in Reducing Ethnic Prejudice Among Adolescents
Source: J Youth Adolesc. 2022 Jul 1;51(10):1970–82. doi: 10.1007/s10964-022-01650-0 (PMC9361236; doi:10.1007/s10964-022-01650-0)
Supplement: Supplementary file 1 — Supplementary Materials [file 10964_2022_1650_MOESM1_ESM.docx]

**SUPPLEMENTAL MATERIALS**

**Table S1**

Means, standard deviations, and correlations among study variables.

|  | *M* | *SD* | 1. | 2. | 3. | 4. | 5. | 6. | 7. | 8. | 9. | 10. | 11. | 12. | 13. | 14. | 15. | 16. | 17. | 18. | 19. |
| --- | --- | --- | --- | --- | --- | --- | --- | --- | --- | --- | --- | --- | --- | --- | --- | --- | --- | --- | --- | --- | --- |
| 1.Age |  |  |  |  |  |  |  |  |  |  |  |  |  |  |  |  |  |  |  |  |  |
| 2.Gender |  |  | -.20^**^ |  |  |  |  |  |  |  |  |  |  |  |  |  |  |  |  |  |  |
| 3.EC T1 | 3.61 | 0.57 | -.05 | .09 |  |  |  |  |  |  |  |  |  |  |  |  |  |  |  |  |  |
| 4.PT T1 | 3.32 | 0.53 | -.07 | .03 | .30^***^ |  |  |  |  |  |  |  |  |  |  |  |  |  |  |  |  |
| 5.AP T1 | 39.70 | 30.93 | .18^**^ | -.01 | -.25^***^ | -.20^**^ |  |  |  |  |  |  |  |  |  |  |  |  |  |  |  |
| 6.CP T1 | 1.70 | 0.58 | .10 | -.00 | -.12 | -.25^***^ | .53^***^ |  |  |  |  |  |  |  |  |  |  |  |  |  |  |
| 7.BP Contact T1 | 1.80 | 0.66 | .06 | -.06 | -.20^**^ | -.23^***^ | .44^***^ | .58^***^ |  |  |  |  |  |  |  |  |  |  |  |  |  |
| 8.BP Helping T1 | 1.80 | 0.58 | .12 | -.17^**^ | -.38^***^ | -.32^***^ | .40^***^ | .51^***^ | .50^***^ |  |  |  |  |  |  |  |  |  |  |  |  |
| 9.EC T2 | 3.54 | 0.56 | -.06 | .14^*^ | .72^***^ | .36^***^ | -.34^***^ | -.25^***^ | -.24^***^ | -.42^***^ |  |  |  |  |  |  |  |  |  |  |  |
| 10.PT T2 | 3.41 | 0.58 | -.11 | .09 | .47^***^ | .61^***^ | -.33^***^ | -.31^**^ | -.22^**^ | -.32^***^ | .62^***^ |  |  |  |  |  |  |  |  |  |  |
| 11.AP T2 | 35.08 | 33.01 | .16^*^ | .04 | -.31^**^ | -.11 | .76^***^ | .54^***^ | .44^***^ | .40^***^ | -.37^***^ | -.31^***^ |  |  |  |  |  |  |  |  |  |
| 12.CP T2 | 1.84 | 0.70 | .09 | -.08 | -.25^***^ | -.09 | .42^***^ | .62^***^ | .47^***^ | .38^***^ | -.31^***^ | -.23^**^ | .52^***^ |  |  |  |  |  |  |  |  |
| 13.BP Contact T2 | 2.00 | 0.75 | .08 | -.10 | -.29^***^ | -.09 | .46^***^ | .56^***^ | .63^***^ | .48^***^ | -.35^***^ | -.25^***^ | .52^***^ | .76^***^ |  |  |  |  |  |  |  |
| 14.BP Helping T2 | 1.95 | 0.64 | .09 | -.23^**^ | -.41^***^ | -.20^**^ | .38^***^ | .43^***^ | .41^***^ | .72^***^ | -.50^***^ | -.33^***^ | .41^***^ | .47^***^ | .57^***^ |  |  |  |  |  |  |
| 15.EC T3 | 3.55 | 0.58 | -.17^**^ | .22^**^ | .63^***^ | .31^***^ | -.28^***^ | -.25^***^ | -.30^***^ | -.42^***^ | .75^***^ | .50^***^ | -.33^***^ | -.29^***^ | -.35^***^ | -.55^***^ |  |  |  |  |  |
| 16.PT T3 | 3.44 | 0.60 | -.10 | .07 | .46^***^ | .58^***^ | -.30^***^ | -.34^***^ | -.23^***^ | -.35^***^ | .59^***^ | .76^***^ | -.32^***^ | -.28^***^ | -.26^***^ | -.41^***^ | .55^***^ |  |  |  |  |
| 17.AP T3 | 39.10 | 31.31 | .16^*^ | -.03 | -.23^**^ | -.11 | .68^***^ | .52^***^ | .37^***^ | .33^***^ | -.35^***^ | -.30^***^ | .80^***^ | .43^***^ | .45^***^ | .33^***^ | -.33^***^ | -.33^***^ |  |  |  |
| 18.CP T3 | 1.90 | 0.64 | .18^**^ | -.18^**^ | -.13^*^ | -10 | .46^***^ | .71^***^ | .51^***^ | .41^***^ | -.25^**^ | -.24^**^ | .54^***^ | .65^***^ | .58^***^ | .48^***^ | -.32^***^ | -.32^***^ | .60^***^ |  |  |
| 19.BP Contact T3 | 1.98 | 0.69 | .14^*^ | -.15^*^ | -.13 | -.03 | .39^***^ | .54^***^ | .51^***^ | .34^***^ | -.26^***^ | -.22^**^ | .46^***^ | .57^***^ | .65^***^ | .52^***^ | -.40^***^ | -.32^***^ | .49^***^ | .65^***^ |  |
| 20.BP Helping T3 | 2.12 | 0.71 | .21^*^ | -.22^**^ | -.29^***^ | -.21^**^ | .28^***^ | .43^***^ | .33^***^ | .56^***^ | -.35^***^ | -.33^***^ | .33^***^ | .37^***^ | .45^***^ | .64^***^ | -.54^***^ | -.41^***^ | .35^***^ | .49^***^ | .55^***^ |

*Note*. Gender: 0 = male, 1 = female. EC = empathic concern; PT = perspective-taking; AP = affective prejudice; CP = cognitive prejudice; BP Contact = Behavioral prejudice, Contact willingness scale; BP Helping = Behavioral prejudice, Helping intentions scale. For the sake of results interpretation, higher levels of contact willingness and helping intentions represent higher behavioral prejudice (i.e., represent lower willingness for contact with and lower intentions to help foreign people).

^*^ *p* < 0.05; ^**^ *p* < 0.01; ^***^ *p* < 0.001.

**Longitudinal Measurement Invariance of Study Variables**

As a preliminary step, configural and metric levels of longitudinal measurement invariance were tested for each variable included in the Cross-Lagged Panel Model. To this end, the configural models function as baseline models and should therefore display a good fit, evaluated based on the following criteria. The Comparative Fit Index (CFI) and the Tucker–Lewis Index (TLI) with values higher than .90 and .95 indicative of an acceptable and very good fit, respectively. The Root Mean Square Error of Approximation (RMSEA) and the Standardized Root Mean Residual (SRMR) with values below .08 and .05 indicative of an acceptable and very good fit, respectively (Byrne, 2012). Additionally, the RMSEA’s 90% confidence interval’s upper bound lower than .10 indicates an acceptable fit of the model (Chen et al., 2008). In order to establish metric invariance (i.e., constraining factor loadings to be equal across time), changes in fit indices from the configural to the metric model were evaluated (e.g., Cheung & Rensvold, 2002). Specifically, a significant Δχ_SB_^2^ (Satorra & Bentler, 2001), and ΔCFI ≥ -.010 supplemented by ΔRMSEA ≥ .015 (Chen, 2007) are indicative of non-invariance. Metric invariance (which is the minimum requirement for cross-lagged panel analyses) was established for all variables included in this study. Results are displayed in Table S2.

**Table S2**

Longitudinal Measurement Invariance of Study Variables.

| Models | Model fit | | | | | |  |  | Model comparisons | | |
| --- | --- | --- | --- | --- | --- | --- | --- | --- | --- | --- | --- |
|  | χ^2^ | df | CFI | TLI | SRMR | RMSEA [90% CI] |  | Models | Δχ_SB_^2^ | ΔCFI | ΔRMSEA |
| Empathic Concern | | | | | | | | | | | |
| Configural (M1) | 208.280 | 165 | .965 | .955 | .046 | .032 [.016, .044] |  |  |  |  |  |
| Metric (M2) | 222.361 | 177 | .963 | .956 | .057 | .031 [.016, .044] |  | M2-M1 | 14.146 (12) | -.002 | -.001 |
| Perspective-taking | | | | | | | | | | | |
| Configural (M1) | 204.382 | 165 | .971 | .963 | .052 | .030 [.013, .043] |  |  |  |  |  |
| Metric (M2) | 221.965 | 177 | .967 | .961 | .064 | .031 [.015, .044] |  | M2-M1 | 18.618 (12) | -.004 | .001 |
| Affective Prejudice | | | | | | | | | | | |
| Configural (M1) | 185.630 | 114 | .973 | .964 | .024 | .049 [.036, .062] |  |  |  |  |  |
| Metric (M2) | 201.146 | 124 | .971 | .964 | .027 | .049 [.036, .061] |  | M2-M1 | 15.205 (10) | -.002 | .000 |
| Cognitive Prejudice | | | | | | | | | | | |
| Configural (M1) | 27.993 | 15 | .990 | .975 | .023 | .058 [.022, .091] |  |  |  |  |  |
| Metric (M2) | 27.156 | 19 | .993 | .988 | .025 | .041 [.000, .073] |  | M2-M1 | 0.735 (4) | .003 | -.017 |
| Behavioral Prejudice - Contact Willingness | | | | | | | | | | | |
| Configural (M1) | 21.985 | 15 | .985 | .964 | .031 | .042 [.000, .078] |  |  |  |  |  |
| Metric (M2) | 21.269 | 19 | .995 | .991 | .033 | .021 [.000, .060] |  | M2-M1 | 0.576 (4) | .010 | -.021 |
| Behavioral Prejudice – Helping Intentions | | | | | | | | | | | |
| Configural (M1) | 5.347 | 15 | 1.00 | 1.00 | .014 | .000 [.000, .000] |  |  |  |  |  |
| Metric (M2) | 18.939 | 19 | 1.00 | 1.00 | .043 | .000 [.000, .054] |  | M2-M1 | 14.328 (4)^**^ | .000 | .000 |

*Note*. M = model; χ^2^ = chi-square; df = degree of freedom; CFI = Comparative Fit Index; TLI = Tucker-Lewis Index; SRMR = Standardized Root Mean Square Residual;

RMSEA = Root Mean Square Error of Approximation; CI = confidence interval; Δ = change in the parameter. * *p* < 0.05; ** *p* < 0.01; *** *p* < 0.001

**Sensitivity Analyses**

As ancillary sensitivity analyses, we checked whether the cross-lagged panel model results significantly changed when accounting for demographic variables which have been previously associated with adolescents’ prejudice (Rekker et al., 2015; Weber, 2019). Specifically, first, we estimated an unconstrained model (M1) with cross-lagged paths between empathic competences and dimensions of ethnic prejudice, controlling for: (a) stability or autoregressive paths (i.e., T1🡪T2, T2🡪T3, T1🡪T3), (b) within-time correlations among all variables (i.e., correlations among variables at T1, and correlated changes of variables at T2 and T3), and (c) the effects of participants’ gender (0 = male, 1 = female) and mothers’ and fathers’ educational level (1 = low, 2 = medium, and 3 = high). This model showed a very good fit (Table S3a). Next, a model (M2) with cross-lagged paths fixed to be equal across waves (i.e., T1🡪T2 paths constrained to be equal to T2🡪T3 paths) was tested and compared against the unconstrained one, and a model (M3) with fixed cross-lagged paths and fixed correlated changes (i.e., within-time correlations at T2 and T3) was tested and compared against M2. Both models included the effects of covariates, which were left unconstrained allowing them to exert potentially different effects on empathic competences and ethnic prejudice measured over time. The model comparison confirmed that model (M3) with fixed cross-lagged paths and fixed correlated changes was not different from M2 and was retained as the most parsimonious solution. Results of the cross-lagged panel model are reported in Table S3b. When compared with results reported in the manuscript, only two differences were found. First, empathic concern was not significantly associated with lower levels of cognitive prejudice over time. Second, cognitive prejudice was not significantly associated with lower perspective-taking abilities over time. Both paths were significant at *p* < .05 in the original model. Therefore, including participants’ gender and their parents’ educational level in the model as covariates did not change the model substantially. Additionally, participants’ gender was significantly associated with empathic concern at T3 and helping intentions at T2, and marginally with cognitive prejudice at T3: female adolescents displayed significantly higher levels of concern and lower levels of behavioral prejudice.

**Table S3a**

Cross-lagged panel model with covariates: Model fit indices and model comparison

| Models | Model fit | | | | | |  | Model comparisons | | | |
| --- | --- | --- | --- | --- | --- | --- | --- | --- | --- | --- | --- |
|  | χ_SB_^2^ | df | CFI | TLI | SRMR | RMSEA  [90% CI] |  | Models | Δχ_SB_^2^ | ΔCFI | ΔRMSEA |
| Unconstrained (M1) | 46.114 | 30 | .993 | .950 | .014 | .046  [.014, .070] |  |  |  |  |  |
| Cross-lagged paths fixed (M2) | 89.235 | 60 | .987 | .955 | .028 | .043  [.022, .061] |  | M2-M1 | 43.260 (30) | -.006 | -.003 |
| Cross-lagged paths and within time correlations fixed (M3) | 123.003 | 75 | .978 | .941 | .036 | .050  [.033, .065] |  | M3-M2 | 31.937 (15)^**^ | -.009 | .007 |

*Note*. M = model; χ_SB_^2^ = Satorra-Bentler scaled chi-square; df = degree of freedom; CFI = Comparative Fit Index; TLI = Tucker-Lewis Index; SRMR = Standardized Root Mean Square Residual; RMSEA = Root Mean Square Error of Approximation; CI = confidence interval; Δ = change in the parameter.

* *p* < 0.05; ** *p* < 0.01; *** *p* < 0.001

**Table S3b**

Standardized results of the cross-lagged panel model with covariates

| Stability paths | T1 → T2 | T2 → T3 | | T1 → T3 |
| --- | --- | --- | --- | --- |
| Empathic concern | .618^***^ | .434^***^ | | .231^**^ |
| Perspective-taking | .500^***^ | .452^***^ | | .188^**^ |
| Affective prejudice | .719^***^ | .601^***^ | | .181^*^ |
| Cognitive prejudice | .504^***^ | .270^**^ | | .362^***^ |
| Behavioral prejudice - Contact willingness | .397^***^ | .211^*^ | | .139^*^ |
| Behavioral prejudice - Helping intentions | .589^***^ | .366^***^ | | .237^**^ |
| Cross-lagged paths | T1 → T2 | | T2 → T3 | |
| Empathic concern → Perspective-taking | .247^***^ | | .220^***^ | |
| Empathic concern → Affective prejudice | -.094^**^ | | -.092^**^ | |
| Empathic concern → Cognitive prejudice | -.075 | | -.071 | |
| Empathic concern → Contact willingness | -.078 | | -.071 | |
| Empathic concern → Helping intentions | -.115^**^ | | -.101^**^ | |
| Perspective-taking → Empathic concern | .057 | | .056 | |
| Perspective-taking → Affective prejudice | .051 | | .056 | |
| Perspective-taking → Cognitive prejudice | .096^*^ | | .102^*^ | |
| Perspective-taking → Contact willingness | .097^*^ | | .100^*^ | |
| Perspective-taking → Helping intentions | .024 | | .024 | |
| Affective prejudice → Empathic concern | -.073 | | -.070 | |
| Affective prejudice → Perspective-taking | -.078^*^ | | -.077^*^ | |
| Affective prejudice → Cognitive prejudice | .132^**^ | | .138^**^ | |
| Affective prejudice → Contact willingness | .142^*^ | | .144^**^ | |
| Affective prejudice → Helping intentions | .047 | | .045 | |
| Cognitive prejudice → Empathic concern | -.005 | | -.005 | |
| Cognitive prejudice → Perspective-taking | -.081 | | -.085 | |
| Cognitive prejudice → Affective prejudice | .065 | | .075 | |
| Cognitive prejudice → Contact willingness | .163^*^ | | .176^*^ | |
| Cognitive prejudice → Helping intentions | .058 | | .060 | |
| Contact willingness → Empathic concern | -.004 | | -.004 | |
| Contact willingness → Perspective-taking | .055 | | .055 | |
| Contact willingness → Affective prejudice | .045 | | .050 | |
| Contact willingness → Cognitive prejudice | .072 | | .077 | |
| Contact willingness → Helping intentions | .021 | | .020 | |
| Helping intentions → Empathic concern | -.125^**^ | | -.131^**^ | |
| Helping intentions → Perspective-taking | -.037 | | -.040 | |
| Helping intentions → Affective prejudice | -.048 | | -.056 | |
| Helping intentions → Cognitive prejudice | .041 | | .047 | |
| Helping intentions → Contact willingness | .152^**^ | | .167^**^ | |
| Covariates | T1 → T2 | | T1 → T3 | |
| Gender → Empathic concern | .073 | | .120^**^ | |
| Gender → Perspective-taking | .040 | | -.002 | |
| Gender → Affective prejudice | .008 | | -.057 | |
| Gender → Cognitive prejudice | -.106 | | -.121^†^ | |
| Gender → Contact willingness | -.069 | | -.057 | |
| Gender → Helping intentions | -.148^**^ | | -.080 | |
| Educational level mother → Empathic concern | .009 | | .009 | |
| Educational level mother → Perspective-taking | .014 | | .010 | |
| Educational level mother → Affective prejudice | .095 | | -.029 | |
| Educational level mother → Cognitive prejudice | .048 | | .027 | |
| Educational level mother → Contact willingness | .019 | | -.010 | |
| Educational level mother → Helping intentions | -.049 | | -.057 | |
| Educational level father → Empathic concern | -.028 | | -.032 | |
| Educational level father → Perspective-taking | .020 | | .035 | |
| Educational level father → Affective prejudice | -.063 | | -.027 | |
| Educational level father → Cognitive prejudice | -.127 | | -.045 | |
| Educational level father → Contact willingness | -.022 | | -069 | |
| Educational level father → Helping intentions | .080 | | .046 | |
| Correlations | T1 | T2 | | T3 |
| Empathic concern ↔ Perspective-taking | .298^***^ | .290^***^ | | .319^***^ |
| Empathic concern ↔ Affective prejudice | -.244^***^ | -.111^**^ | | -.126^**^ |
| Empathic concern ↔ Cognitive prejudice | -.113 | -.126^*^ | | -.140^*^ |
| Empathic concern ↔ Contact willingness | -.189^**^ | -.196^***^ | | -.183^***^ |
| Empathic concern ↔ Helping intentions | -.372^***^ | -.307^***^ | | -.265^***^ |
| Perspective-taking ↔ Affective prejudice | -.195^**^ | -.123^**^ | | -.153^**^ |
| Perspective-taking ↔ Cognitive prejudice | -.245^***^ | -.111 | | -.135^*^ |
| Perspective-taking ↔ Contact willingness | -.228^***^ | -.162^**^ | | -.164^**^ |
| Perspective-taking ↔ Helping intentions | -.325^***^ | -.178^***^ | | -.167^***^ |
| Affective prejudice ↔ Cognitive prejudice | .525^***^ | .259^***^ | | .326^***^ |
| Affective prejudice ↔ Contact willingness | .436^***^ | .216^***^ | | .227^***^ |
| Affective prejudice ↔ Helping intentions | .406^***^ | .184^**^ | | .180^**^ |
| Cognitive prejudice ↔ Contact willingness | .580^***^ | .480^***^ | | .495^***^ |
| Cognitive prejudice ↔ Helping intentions | .524^***^ | .244^***^ | | .234^***^ |
| Contact willingness ↔ Helping intentions | .499^***^ | .392^***^ | | .313^***^ |

*Note*. For the sake of results interpretation, higher levels of contact willingness and helping intentions represent higher behavioral prejudice (i.e., represent lower willingness for contact with and lower intentions to help foreign people).

Results highlighted in grey are those that differ from the main model reported in the manuscript (in the main model these paths were marginally significant, while in the current model with covariates they did not reach significance anymore).

^*^ *p* < 0.05; ^**^ *p* < 0.01; ^***^ *p* < 0.001; ^†^ *p* = 0.05
